# Voriconazole and posaconazole therapeutic drug monitoring: a retrospective study

**DOI:** 10.1186/s12941-017-0235-8

**Published:** 2017-09-11

**Authors:** Whitley M. Yi, Kelly E. Schoeppler, Jaclyn Jaeger, Scott W. Mueller, Robert MacLaren, Douglas N. Fish, Tyree H. Kiser

**Affiliations:** 1 University of Colorado, Skaggs School of Pharmacy and Pharmaceutical Sciences, 12850 E Montview Blvd, Aurora, CO 80045 USA; 2 Department of Pharmacy, University of Colorado Hospital, 12605 E 16th Ave, Aurora, CO 80045 USA; 3 Department of Clinical Pharmacy, University of Colorado, Skaggs School of Pharmacy and Pharmaceutical Sciences, 12850 E Montview Blvd, Aurora, CO 80045 USA; 4 Department of Clinical Pharmacy, University of Colorado Anschutz Medical Campus, 12850 E Montview Blvd, C238, Aurora, CO 80045 USA

**Keywords:** Voriconazole, Posaconazole, Therapeutic drug monitoring, Invasive fungal disease

## Abstract

**Background:**

Therapeutic drug monitoring (TDM) aims to minimize the clinical impact of posaconazole and voriconazole pharmacokinetic variability. However, its benefits on clinical outcomes are still being defined. Additionally, TDM data are limited for posaconazole IV and delayed-release tablet formulations among specific patient populations, including critically ill. The aim of this study was to determine the percentage of therapeutic posaconazole and voriconazole drug levels across all formulations in a real-world clinical setting and elucidate factors affecting attainment of target concentrations.

**Methods:**

This study was a retrospective cohort study conducted at the University of Colorado Hospital between September 2006 and June 2015 that evaluated patients who received posaconazole or voriconazole TDM as part of routine care.

**Results:**

Voriconazole (n = 250) and posaconazole (n = 100) levels were analyzed from 151 patients. Of these, 54% of voriconazole and 69% of posaconazole levels were therapeutic. For posaconazole, 14/38 (37%), 28/29 (97%) and 27/33 (82%) levels were therapeutic for the oral suspension, IV, and delayed-release tablet, respectively. Intravenous and delayed-release tablet posaconazole were 20 fold *(p* < 0.01) and sevenfold (*p* = 0.002) more likely than the oral suspension to achieve a therapeutic level. Subsequent levels were more likely to be therapeutic after dose adjustments (OR 3.31; 95% CI 1.3–8.6; *p* = 0.02), regardless of timing of initial non-therapeutic level. In a multivariable logistic regression analysis, no characteristics were independently predictive of therapeutic voriconazole levels and only absence of H2RA/PPI use was independently predictive of therapeutic posaconazole levels. There was no correlation between survival and therapeutic drug levels for either voriconazole (*p* = 0.67) or posaconazole (*p* = 0.50).

**Conclusions:**

A high percentage of drug levels did not achieve TDM targets for voriconazole and posaconazole oral suspension, supporting the need for routine TDM for those formulations. The utility of TDM for the IV and delayed-release tablet formulations of posaconazole is less apparent.

## Background

Posaconazole and voriconazole are triazoles with broad spectrum antifungal activity; however, their pharmacokinetics pose a therapeutic challenge. Voriconazole is used first line for invasive aspergillosis (IA), while posaconazole is indicated as salvage therapy and prophylaxis for IA [1] and has activity against Mucorales [2]. Both antifungals exhibit highly variable inter- and intra-patient pharmacokinetics [3, 4], prompting development of newer dosage forms of posaconazole aimed at minimizing pharmacokinetic variability. Numerous factors have been associated with variability in voriconazole and posaconazole plasma levels; examples include altered intestinal absorption, drug interactions, diarrhea, chemotherapy, age, and weight [5–9].

For both voriconazole and posaconazole, the relationship between efficacy and drug exposure has been established [7, 10, 11]. A voriconazole plasma level of 1.0–5.5 or 1.0–6.0 μg/ml is generally recommended as the goal range for improved outcomes and minimized toxicities [11, 12], and a threshold of >0.5–0.7 μg/ml has been recommended as a therapeutic posaconazole concentration [13, 14]. Given high risk of mortality for invasive fungal disease and the inter- and intra-patient variability in posaconazole and voriconazole pharmacokinetics, therapeutic drug monitoring (TDM) is a strategy employed to optimize drug therapy. There has been an increase in TDM practices for antifungal agents [15], especially with voriconazole [16]. Additionally, the Infectious Diseases Society of America (IDSA) guidelines for aspergillosis recommend steady-state TDM for azole-based treatment of invasive aspergillosis [1]. However, as noted in the IDSA guidelines, more studies are needed to look at the benefit of TDM specifically for the intravenous and delayed-release tablet formulations of posaconazole, which has not been well-established.

A predominant focus of voriconazole and posaconazole TDM studies has been on hematologic cancer patient populations. Few studies have assessed other patient populations, such as the critically ill, who may have the lowest rates of attaining therapeutic drug levels of posaconazole [17]. TDM practices can be different in the intensive care unit (ICU) as opposed to other less emergent settings, as seen with the high rate of drug levels drawn on day 1 or 2 of therapy [18]. While guidelines recommend waiting until steady state for first TDM measurement [1], studies have not evaluated whether this is strictly adhered to in routine practice and what the implications on efficacy are if it is not.

This study took a broad look at patients receiving TDM for voriconazole and posaconazole, including patients in the critical care setting. The aim of this study was to determine the percentage of therapeutic posaconazole and voriconazole drug levels across all available formulations in a real-world clinical setting, and elucidate patient characteristics that affect ability to attain target plasma drug concentrations.

## Methods

### Study design

This study was a retrospective cohort study conducted at the University of Colorado Hospital between September 2006 and June 2015. The study was approved by the Colorado Multiple Institutional Review Board.

### Patients

Patients were identified through the University of Colorado Hospital’s Electronic Health Record. Patients were included in the study if they had received voriconazole or posaconazole and had a drug level drawn for TDM as part of routine clinical care. Exclusion criteria included patients older than 89 or younger than 18 years, patients who were incarcerated, or patients who were pregnant.

### Drug levels

All drug levels were drawn as part of routine care, with the decision to perform TDM based on the care team. Voriconazole trough drug levels were designated as therapeutic if they were >1 and <5.5 μg/ml. Posaconazole trough drug levels were designated as therapeutic if they were >0.7 μg/ml [14]. All drug levels were evaluated via HPLC analysis at the Clinical Laboratory Improvement Amendments (CLIA) certified National Jewish Clinical Pharmacology lab (Denver, Colorado). The medical team received all results in real time for dose adjustments to be made if needed.

For analysis, all drug levels were treated as individual data points unless indicated otherwise. For voriconazole and posaconazole, steady state was defined as day 5 of therapy or later, whether after the initiation of therapy or a change in dose. When performing analyses between patients, rather than individual drug levels, patients were categorized as therapeutic or not based on the mean of their drug concentration measurements, as reported in previous TDM studies [10, 19]. If a patient had alternated between therapy on voriconazole and posaconazole, the mean of each drug was evaluated separately, and these patients were included in both the voriconazole and posaconazole treatment groups for analysis.

### Interacting medications

For voriconazole, interacting medications included amiodarone, diltiazem, erythromycin, phenobarbital, phenytoin, rifampin, and valproic acid. Posaconazole was considered to have the same potential interactions with the addition of metoclopramide.

### Invasive fungal disease assessment

Invasive fungal disease (IFD) was classified as possible, probable, or proven per the European Organization for Research and Treatment of Cancer/Invasive Fungal Infections Cooperative Group and the National Institute of Allergy and Infectious Diseases Mycoses Study Group (EORTC/MSG) Consensus Group [20] with the following modifications: if a patient met mycological criteria and clinical criteria, the infection was classified as probable, even in the absence of a host factor. This was to account for immunocompromising patient factors outside those listed by EORTC/MSG as host factors. Patients who came in with a diagnosed IFD from an outside hospital were classified as probable. Patients with criteria for a proven infection from a previous hospital admission were classified as proven if still undergoing active treatment for the same infection. Immunocompromised status was defined as neutropenia, allogeneic stem cell transplant, solid organ transplant, prolonged use of corticosteroids, or T-cell immunosuppressive therapy.

Treatment failure was defined as discontinuation due to an adverse event, a breakthrough infection during prophylaxis, continued positive cultures, or worsening or persistent positive imaging for proven or probable IFD. Patient follow-up for clinical outcomes was assessed only up until patient discharge. Mortality was determined based on patient survival to hospital discharge. If patients had more than one hospital admission, the most recent admission was used in categorizing survival or treatment failure.

### Primary outcome

The primary outcome evaluated the percentage of voriconazole and posaconazole plasma trough levels within goal.

### Secondary outcomes

The secondary outcomes for the study were the percentage of patients unable to achieve a mean therapeutic voriconazole or posaconazole plasma concentration; correlation of clinical outcomes and adverse events with patient mean plasma concentration; correlation of concomitant H2 receptor antagonist/proton pump inhibitor (H2RA/PPI) use and dosage formulation with achievement of therapeutic plasma levels; comparison of the percentage of therapeutic posaconazole and voriconazole levels achieved among specific patient populations; percentage of posaconazole and voriconazole TDM measurements that were drawn prior to steady-state; the percentage of therapeutic drug levels achieved after dose modification; and the correlation between pre and post steady-state plasma concentrations without a dose adjustment.

### Statistical analysis

Categorical data were compared via the Fisher exact test. Continuous data were evaluated using the Wilcoxon rank sum or the Student *t*-test, depending on data distribution. A matched pair analysis was used to evaluate the relationship between levels pre and post steady-state. Multivariable logistic regression was utilized to evaluate the impact of patient characteristics and drug dosages on achieving therapeutic drug concentrations. Variables with a p value <0.1 in the univariate analysis were included in the model (weight, TDD, mg/kg/dose, mg/kg/day, immunosuppression, ICU, and concomitant H2RA/PPI use). JMP Pro version 12.0.1 (SAS, Cary, North Carolina) was utilized to perform all analyses. A p value <0.05 was considered significant.

## Results

### Study population

A total of 350 drug levels were analyzed from 151 patients. Of the 151 patients, 79 (48%) were male, 93 (62%) were Caucasian, 68 (45%) were critically ill, and 61 (40%) were transplant recipients (Table 1). Median age was 50 years (IQR 33–60 years) (Table 1). For patients with identified fungal pathogens, *Aspergillus* (37 of 103; 36%) was the most common, followed by *Candida* (34 of 103; 33%) (Table 1). There were 122 patients who received voriconazole TDM and 38 who received posaconazole TDM. Nine patients were counted in both drug groups, as they had sequentially taken voriconazole and posaconazole and had TDM performed on both.

**Table 1:** Patient characteristics

Characteristics	Overall (n = 151)	Voriconazole (n = 122)	Posaconazole (n = 38)
Male	79 (48)	60 (49)	22 (58)
Age (year) (median [IQR])	50 (33–60)	50 (33–60)	48 (33–60)
Weight^a^ (kg) (median [IQR])	70.8 (59.8–82.6)	69.7 (58.6–82.1)	74.0 (63.4–84.0)^b^
Race and ethnicity			
Caucasian	93 (62)	77 (63)	21 (55)
African American	10 (7)	7 (6)	3 (8)
American India or Alaskan native	3 (2)	3 (2)	1 (3)
Asian	3 (2)	3 (2)	0
Hispanic	19 (12)	12 (10)	8 (21)
Unknown	23 (15)	20 (17)	5 (13)
Critically ill	68 (45)	57 (47)	16 (42)
Transplant status			
Solid organ recipients	23 (15)	18 (15)	7 (18)
Bone marrow recipients	38 (25)	33 (27)	10 (26)
Comorbidities			
Cystic fibrosis	18 (12)	17 (14)	1 (3)
Type of infection			
Prophylaxis	9 (6)	5 (4)	4 (11)
Possible	32 (21)	27 (22)	10 (26)
Probable	57 (38)	51 (42)	6 (16)
Proven	53 (35)	39 (32)	18 (47)
Identified organism (n = 103)		(n = 82)	(n = 21)
*Candida* spp.		31 (38)	3 (14)
*Aspergillus* spp.		34 (41)	3 (14)
*Mucor*		0	11 (52)
*Scedosporium* spp.		7 (9)	0
*Cryptococcus*		1 (1)	1 (4)
Other		9 (11)	3 (14)
Outcomes			
Achieved a mean therapeutic level		74 (61)	22 (58)
Survived to hospital discharge	109 (72)	90 (74)	26 (68)
Treatment failure	39 (26)	33 (27)	7 (18)

Data are presented as n (%) or median (interquartile range)

*IQR* interquartile range

^a^Mean weight for each individual was used if there was >1 value

^b^n = 37 (weight for one individual unavailable)

### Drug levels

**Fig. 1:**
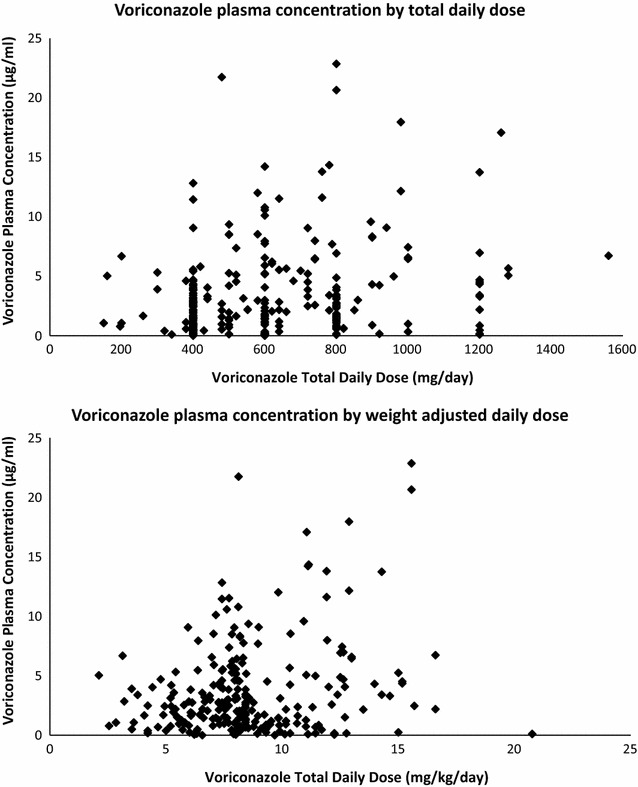
Voriconazole dose and resultant plasma concentration

**Fig. 2:**
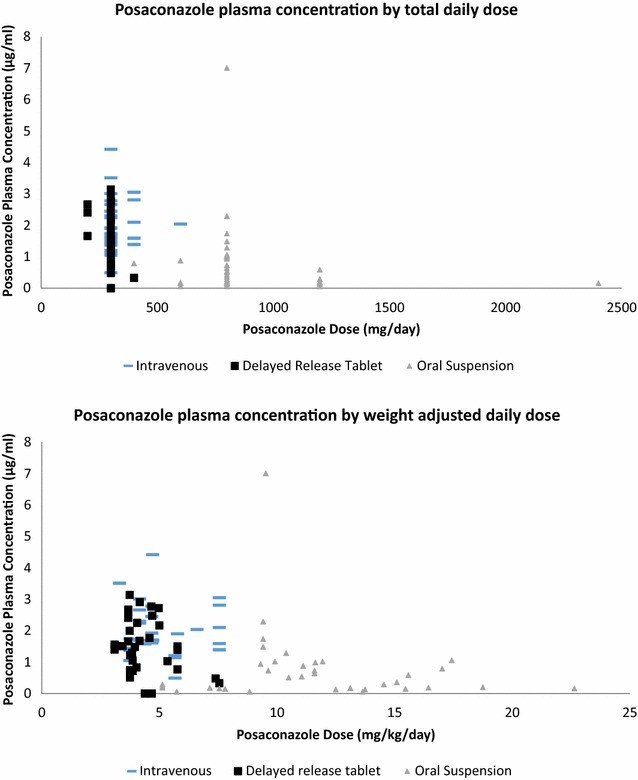
Posaconazole dose and resultant plasma concentration

Of the 350 drug levels, 250 voriconazole and 100 posaconazole trough levels were analyzed. Figure 1 displays voriconazole drug concentration by TDD (mg/day) and weight-normalized TDD (mg/kg/day). The median voriconazole plasma concentration was 2.43 μg/ml (Table 2) and the median posaconazole plasma concentration was 1.26 μg/ml (Table 3). Figure 2 displays posaconazole drug concentration by TDD (mg/day) and weight-normalized TDD (mg/kg/day) and type of formulation. For voriconazole, 134 of 250 (54%) levels were in the therapeutic range. Of the 116 non-therapeutic voriconazole levels, 65 were subtherapeutic and 51 were supratherapeutic. For posaconazole, 69 of 100 (69%) levels were >0.7 μg/ml. The mean number of drug levels drawn per patient was 2.3 levels. For patients taking voriconazole, 74 (61%) patients maintained an average drug level that was in the therapeutic range, and for posaconazole, 22 (58%) patients maintained an average drug level that was therapeutic (Table 1).

**Table 2:** Voriconazole

Overall (n = 250)			
Drug concentration **(**μg/ml**)** (median [IQR])		2.43 (0.94–4.90)	
Comparison by drug level	Therapeutic drug level (n = 134)	Non-therapeutic drug level (n = 116)	*p*
PK parameters			
Drug concentration (μg/ml) (median [IQR])	2.6 (1.66–3.87)	0.90 (0.45–7.97)	0.10
Total daily dose (mg) (median [IQR])	552 (400–800)	600 (405–800)	0.01
mg/kg/dose (median [IQR])	3.95 (3.3–4.4)	4.24 (3.7–5.5)	0.01
mg/kg/day (median [IQR])	7.76 (6.4–8.7)	8.48 (7.5–11.0)	<0.01
Weight (median [IQR])	70.8 (61.0–84.3)	75.5 (58.6–88.75)	0.65
Subtherapeutic (n = 65)		72 (56.5–94)	0.90
Supratherapeutic (n = 51)		86.1 (76.1–102.7)	0.36
Concomitant interacting medications	13 (10)	15 (13)	0.43
Concomitant H2RA/PPI	113 (85)	92 (79)	0.25
PK parameters—initial dose only	(n = 66)	(n = 57)	
mg/kg/dose (median [IQR])	3.67 (2.9–4.2)	4.03 (3.6–4.7)	0.01
mg/kg/day (median [IQR])	7.34 (5.8–8.4)	8.07 (7.1–9.3)	0.01
Weight (median [IQR])	69.0 (60.5–82)^a^	74.0 (58.1–85.5)	0.69
Subtherapeutic (n = 29)		64.4 (49.5–79.3)	0.11
Supratherapeutic (n = 28)		80.5 (68.1–88.8)	0.054
Patient characteristics			
Critically ill	54 (40)	47 (41)	1.00
Solid organ transplant recipients	15 (11)	17 (15)	0.45
Bone marrow transplant recipients	41 (31)	28 (24)	0.26
Cystic fibrosis	17 (13)	11 (9)	0.55
Immunocompromised	90 (67)	81 (70)	0.68
Outcomes			
Length of stay (days) (median [IQR])	31 (14–56)	27 (8–41)	0.12
Adverse drug event requiring discontinuation	1 (0.7)	8 (7)	0.01
Treatment failure	35 (26)	44 (38)	0.06

Comparison by patient	Therapeutic mean drug level (n = 74)	Non-therapeutic mean drug level (n = 48)	
Outcomes			
Survive to hospital discharge (n = 122)	56 (76)	34 (71)	0.67
Treatment failure	12 (16)	21 (44)	<0.01
Length of stay (days) (median [IQR])	22 (11–37)	21 (7–37)	0.25

Data are presented as n (%) or median (interquartile range)

*IQR* interquartile range, *PK* pharmacokinetic

^a^n = 67

**Table 3:** Posaconazole

Overall (n = 100)			
Drug concentration (μg/ml) (Median [IQR])		1.26 (0.50–1.91)	
Comparison	Therapeutic drug level (n = 69)	Non-therapeutic drug level (n = 31)	*p*
PK parameters			
Drug concentration (μg/ml) (median [IQR])	1.61 (1.17–2.30)	0.19 (0.15–0.48)	<0.01
Total daily dose (mg) (median [IQR])	300 (300–400)	800 (600–1200)	<0.01
mg/kg/dose (median [IQR])	4.03 (3.3–4.9)	4.08 (2.5–5.5)	0.75
mg/kg/day (median [IQR])	4.68 (3.8–7.5)	9.67 (5.5–13.9)	<0.01
Weight (median [IQR])	72 (55.3–80.0)	77 (64.0–88.6)	0.05
Concomitant interacting medication	13 (19)	8 (26)	0.44
PK parameters—initial dose only	(n = 21)	(n = 17)	
mg/kg/dose (median [IQR])	4.16 (3.6–5.2)	4.38 (2.5–5.4)	0.54
mg/kg/day (median [IQR])	5.67 (4.0–9.6)	10.49 (5.4–14.4)	0.04
Weight (median [IQR])	74 (59.0–84.5)	77 (64.0–87.7)	0.36
Patient characteristics			
Critically ill	34 (49)	16 (52)	1.00
Solid organ transplant recipients	17 (25)	4 (13)	0.29
Bone marrow transplant recipients	14 (20)	12 (39)	0.08
Cystic fibrosis	1 (1)	1 (3)	0.53
Immunocompromised^a^	41 (59)	25 (81)	0.04
Outcomes			
Length of stay (days) (median [IQR])	27 (17–48)	43 (36-76)	<0.01
Treatment failure	13 (18)	9 (30)	0.30

Comparison by patient	Therapeutic mean drug level (n = 22)	Non-therapeutic mean drug level (n = 16)	
Outcomes			
Survive to hospital discharge (n = 38)	14 (64)	12 (75)	0.50
Treatment failure	4 (18)	3 (18)	1.00
Length of stay (days) (median [IQR])	23 (8–36)	40 (35–57)	<0.01

Data are presented as n (%) or median (interquartile range)

*IQR* interquartile range

^a^Neutropenia, allogeneic stem cell transplant, solid organ transplant, prolonged use of corticosteroids, or T-cell immunosuppressive therapy

Overall, 126 (36%) levels were drawn prior to steady state. For voriconazole, 96 (38%) levels were pre-steady state and 30 (30%) for posaconazole. Of these early levels, 42 (43%) (19 subtherapeutic; 23 supratherapeutic) and 12 (40%) were non-therapeutic for voriconazole and posaconazole, respectively. The mean ± SD in-hospital length of therapy among those who were discharged on an antifungal or did not survive to discharge was 20.0 ± 18.0 and 18.6 ± 18.5 days for voriconazole and posaconazole, respectively.

### Pharmacokinetic parameters and patient characteristics

When comparing therapeutic versus non-therapeutic levels of combined voriconazole and posaconazole, type of organism was statistically different between groups (*p* < 0.01), while the only patient characteristic significantly different between groups was race/ethnicity (*p* < 0.01) (Table 4). When analyzing voriconazole and posaconazole levels individually, no patient characteristics were significantly correlated with achieving a therapeutic drug level for voriconazole. However, among non-therapeutic posaconazole drug levels, immunocompromised status (*p* = 0.04) and bone marrow transplant recipient status *(p* = 0.08) were more common (Table 3). Concomitant H2RA or PPI use was present with 28 of 38 (74%) posaconazole oral suspension levels and 19 of 33 (58%) posaconazole delayed-release tablet levels (Table 5). Both formulations had a higher proportion of concomitant H2RA/PPI use among the non-therapeutic drug level group compared to the proportion in the therapeutic drug level group (Table 5). The odds ratio of achieving a therapeutic level with posaconazole suspension and concomitant H2RA/PPI use was 0.07 (95% CI 0.01–0.41; *p* < 0.01).

**Table 4:** Drug level comparison

	Therapeutic drug level (n = 203)	Non-therapeutic drug level (n = 147)	*p*
Antifungal medication			
Voriconazole	134 (66)	116 (79)	<0.01
Posaconazole	69 (34)	31 (21)	
Timing of level			
Pre-steady-state	72 (35)	54 (37)	0.82
Steady-state	131 (65)	93 (63)	
Male	98 (48)	84 (57)	0.11
Weight (kg) (median [IQR])	71.0 (60.3–83.0)	76.2 (59.1–88.3)^a^	0.13
Age (year) (median [IQR])	49 (36–61)	47 (33–60)	0.15
Race and ethnicity			<0.01
Caucasian	135 (67)	89 (61)	
African American	20 (10)	6 (4)	
American India or Alaskan native	4 (2)	6 (4)	
Asian	6 (3)	0	
Hispanic	22 (11)	17 (12)	
Unknown	16 (8)	29 (20)	
Critically Ill	88 (43)	63 (43)	1.00
Transplant status			
Solid organ recipients	32 (16)	21 (14)	0.76
Bone marrow recipients	55 (27)	40 (27)	1.00
Comorbidities			
Cystic fibrosis	18 (9)	12 (8)	0.85
Gastrointestinal	46 (23)	25 (17)	0.23
Type of infection			
Prophylaxis	15 (7)	7 (5)	0.38
Possible	33 (16)	32 (22)	
Probable	58 (29)	46 (31)	
Proven	97 (48)	62 (42)	
Organism (n = 245)			
*Candida* spp.	29 (20)	38 (37)	<0.01
*Aspergillus* spp.	48 (33)	34 (34)	
*Mucor*	31 (21)	7 (7)	
*Scedosporium* spp.	18 (12)	13 (13)	
*Cryptococcus*	7 (5)	0	
Other	13 (9)	9 (9)	

Data are presented as n (%) or median (interquartile range)

*IQR* interquartile range

^a^n = 146 (weight unavailable for one drug level)

**Table 5:** Posaconazole and concomitant PPI/H2RA use

Concomitant use of PPI or H2RA	Therapeutic (n = 46)	Non-therapeutic (n = 29)	*p*
Intravenous	(n = 28)	(n = 1)	
With PPI/H2RA	27 (96)	1 (100)	1.00
No PPI/H2RA	1 (4)	0	
Oral Suspension	(n = 14)	(n = 24)	
With PPI/H2RA	6 (43)	22 (92)	<0.01
No PPI/H2RA	8 (57)	2 (8)	
Delayed-release tablet	(n = 27)	(n = 6)	
With PPI/H2RA	13 (48)	6 (100)	0.03
No PPI/H2RA	14 (52)	0	

Data are presented as n (%)

Patient weight, total daily dose (TDD), and weight-normalized TDD (in mg/kg/day) were significantly correlated with achievement of goal posaconazole drug level (Table 3). However, when looking just at initial levels, only weight-normalized TDD (in mg/kg/day) was significantly correlated with achievement of goal posaconazole level. For voriconazole, weight-normalized TDD (in mg/kg/day), mg/kg/dose, and TDD were significantly correlated with achievement of voriconazole goal drug level, regardless of looking at all levels or just initial levels (Table 2). Weight was not significantly correlated with voriconazole therapeutic drug levels. This was true for both subtherapeutic and supratherapeutic voriconazole levels (Table 2).

In a multivariable logistic regression analysis, using factors shown to be statistically significant through univariate analysis, only concomitant H2RA/PPI use was an independent determinant of a non-therapeutic posaconazole drug level (*p* = 0.04) (Table 6). Analyzing the same parameters, no factors were shown to be independent determinants for achievement of a therapeutic voriconazole drug level (Table 6).

**Table 6:** Variables independently associated with therapeutic drug levels

Variable	Multivariable logistic regression estimate	*p*	OR (95% CI)
Voriconazole			
Total daily dose (mg)	0.001	0.64	
mg/kg/dose	−0.79	0.33	
mg/kg/day	0.44	0.29	
Weight	−0.006	0.78	
Concomitant H2RA/PPI	0.21	0.23	1.53 (0.77–3.09)
Critically ill	−0.06	0.67	0.88 (0.51–1.53)
Immunocompromised	0.002	0.99	1.003 (0.56–1.79)
Posaconazole			
Total daily dose (mg)	0.009	0.07	
mg/kg/dose	−0.076	0.74	
mg/kg/day	−0.16	0.62	
Weight	−0.015	0.65	
Concomitant H2RA/PPI	−1.20	0.04	0.09 (0.007–0.75)
Critically ill	−0.34	0.43	0.05 (0.07–2.45)
Immunocompromised	−0.16	0.73	0.73 (0.11–4.53)

*OR* odds ratio, *CI* confidence interval

### Efficacy and adverse events

Achieving target mean drug concentration did not significantly affect survival to hospital discharge for either antifungal, however, there was a 37% reduction in length of hospital stay for the therapeutic posaconazole level group (*p* < 0.01) (Table 3). This relationship remained significant when accounting for patients who did not survive to hospital discharge (*p* < 0.01). Additionally, there was a 42% reduction in treatment failure for patients able to maintain a therapeutic mean voriconazole drug level (*p* < 0.01) (Table 2). Overall, nine voriconazole levels were associated with an adverse drug event (ADE) that led to drug discontinuation. The reported ADEs were hallucinations (n = 4), elevated liver enzymes (n = 2), hyponatremia (n = 1), renal insufficiency and altered mental status (n = 1), and intolerance (n = 1). Non-therapeutic drug levels were greater than 9 times more likely to result in an ADE requiring drug discontinuation (*p* = 0.01) (Table 2). There were only three ADEs reported for posaconazole, all in the therapeutic drug group, with only one leading to discontinuation.

### Drug formulation

Voriconazole dosage formulation was not significantly correlated to achieving therapeutic drug levels (*p* = 0.90) (Table 7). On the other hand, there was a statistically significant difference between posaconazole dosage formulation and achieving therapeutic drug level (*p* < 0.01). For the oral suspension, 14 of 38 levels (37%) were therapeutic, while 28 of 29 (94%) and 27 of 33 (82%) were therapeutic for the intravenous and delayed release tablet, respectively (Table 7). When only comparing IV to PO posaconazole (all formulations), the IV formulation was 20 times more likely to have a therapeutic drug level (OR 20.5; 95% CI 2.64–159; *p* < 0.01). When specifically comparing the suspension to the delayed-release tablet, the delayed-release tablet was associated with a higher chance of achieving therapeutic levels (OR 7.7; 95% CI 2.6–23.2; *p* < 0.01).

**Table 7:** Comparison of dosage formulations

Formulation	Drug concentration (μg/ml)	*p*
Posaconazole		
Delayed-release tablet (median [IQR])	1.48 (0.76–2.21)	<0.01
IV (median [IQR])	1.74 (1.47–2.56)	
Oral suspension (median [IQR])	0.40 (0.17–0.95)	
Voriconazole		
IV (median [IQR])	3.31 (1.41–5.70)	<0.01
PO (median [IQR])	1.78 (0.84–4.00)	

	Therapeutic drug level	Non-therapeutic drug level	OR (95% CI)	*p*
Posaconazole	(n = 69)	(n = 31)		
Delayed-release tablet	27/33 (82%)	6/33 (18%)	7.7 (2.6–23.2)^a^	<0.01
IV	28/29 (97%)	1/29 (3%)	48 (5.9–392.3)^a^	<0.01
Oral suspension	14/38 (37%	24/38 (63%)		
Voriconazole	(n = 134)	(n = 116)		
IV (no. [%])	56 (42)	50 (43)	1.06 (0.64–1.74)	0.90
PO (no. [%])	78 (58)	66 (57)		

*IQR* interquartile range, *OR* odds ratio, *CI* confidence interval

^a^In reference to posaconazole oral suspension

### Dose adjustments

There were 79 non-therapeutic drug levels in which the patient had a subsequent level measured. Only 41% of the time did the subsequent level reflect a dose adjustment. Dose adjustments to non-therapeutic levels were associated with a statistically significant increase in achieving a subsequent therapeutic drug level (OR 3.31; 95% CI 1.3–8.6; *p* = 0.02), but did not correlate to increased survival to hospital discharge (*p* = 1.00) (Table 8). Of the 79 non-therapeutic levels, 32 were drawn prior to steady-state. When looking at these levels alone, dose adjustments were not statistically correlated with achievement of a subsequent therapeutic level (*p* = 0.68). However, the relationship was statistically significant when the initial non-therapeutic drug level was ≥3 days into therapy (*p* = 0.049) (Table 8).

**Table 8:** Analysis of patients with ≥2 drug levels

Comparison action taken on an initial non-therapeutic drug level	Survive to hospital discharge (n = 59)	Did not survive to hospital discharge (n = 20)	OR	*p*
A dose adjustment was made	24 (41)	8 (40)	1.03 (0.4–2.9)	1.00
No dose adjustment was made	35 (59)	12 (60)		

	Therapeutic drug level	Non-therapeutic drug level	OR (95% CI)	*p*
Overall	(n = 29)	(n = 50)		
A dose adjustment was made (no. [%])	17 (59)	15 (30)	3.31 (1.3–8.6)	0.02
No dose adjustment was made (no. [%])	12(41)	35 (70)		
First level prior to steady state^a^	(n = 9)	(n = 23)		
Dose adjustment made (no. [%])	4 (44)	7 (30)	1.83 (0.4–8.9)	0.68
No dose adjustment made (no. [%])	5 (56)	16 (70)		
First level at steady state^a^	(n = 20)	(n = 27)		
Dose adjustment made (no. [%])	13 (65)	8 (30)	4.41 (1.3–15.2)	0.02
No dose adjustment made (no. [%])	7 (35)	19 (70)		
First level measured after ≥3 days	(n = 28)	(n = 44)		
Dose adjustment made (no. [%])	16 (57)	14 (32)	2.86 (1.1–7.6)	0.049
No dose adjustment made (no. [%])	12 (43)	30 (68)		

*OR* odds ratio, *CI* confidence interval

^a^Stead state defined as ≥5 days of therapy

### Timing of drug levels

In a matched pair analysis, there were 29 pairs of levels (voriconazole = 20; posaconazole = 9) with the first level drawn prior to steady state and the second level at steady state in which no dose adjustment was made. The pre-steady state concentrations were predictive of steady state values, as there was no statistical difference between the pre and post steady state levels, with the mean difference in voriconazole levels being −0.509 (95% CI −1.21–0.19; *p* = 0.10) and the mean difference in posaconazole levels being −0.25 (95% CI −0.64–0.14; *p* = 0.34).

## Discussion

The main finding of the study was the high percentage of voriconazole and posaconazole levels not at goal. Forty-six percent of all voriconazole levels measured were outside the therapeutic range, and 39% of patients had an average voriconazole level also outside that range. Posaconazole had a lower percentage of total levels not at goal (31%) but a higher percentage (42%) of patients whose mean plasma level remained non-therapeutic. A possible explanation for the higher percentage of posaconazole patients unable to achieve a mean therapeutic level compared to voriconazole could be the lower rate of TDM in routine practice for posaconazole compared to voriconazole [15], which was a trend also seen at the site of this study. In addition to the potential inexperience in posaconazole TDM-based dose adjustments, the strategies needed for each formulation vary, and evidence has shown previously recommended dose adjustment strategies [14] to be less effective [21]. Even with optimizing posaconazole oral suspension by increasing frequency over dose, however, evidence has not been able to demonstrate a statistically significant difference between median plasma concentrations at baseline and after dose adjustments [21]. Overall, these data demonstrate the challenges of posaconazole TDM and appear to suggest a greater difficultly in successful posaconazole TDM compared to voriconazole TDM.

The 46% of non-therapeutic voriconazole levels was a little higher than reported in the majority of other studies, which ranged from 23 to 37% [9, 18, 22–25]. To our knowledge, only three studies reported percentages greater than 40% [26–28]. The large number of early levels in this study, however, may help explain the higher proportion of sub-therapeutic levels. When looking at posaconazole levels reported in other studies, the rate of not achieving optimal plasma concentrations varied widely from 20% [29] to 90% [30]; however, a majority of the studies report percentages in the range of 20–50% [6, 7, 21, 31–34]. It can be difficult to compare percentages of therapeutic drug measurements across studies due to the differing therapeutic threshold cut-offs, TDM practices, and patient populations at each site. Overall though, it demonstrates that the increase in practicing routine TDM does not appear to translate yet to achievement of higher percentages of therapeutic drug levels.

While other studies have demonstrated correlations between drug level variation and patient characteristics [19], our study looked specifically at correlation between patient characteristics and ability to obtain a therapeutic drug level, not merely a statistical difference in levels. Surprisingly, many of the patient characteristics, such as cystic fibrosis and age [9, 35], that have been associated with drug level variations in either voriconazole or posaconazole in previous studies were not shown to significantly correlate with a therapeutic or non-therapeutic drug level in the current study. For some of the patient characteristics represented by a small sample size, such as cystic fibrosis, the potential for type II error should be considered. However, overall, the data suggests that the effects of patient characteristics on drug level might be more subtle than suggested and potentially less predictive in determining which patients might need preemptive dose adjustment or are more likely to not achieve goal plasma concentrations.

When evaluating patient characteristics for combined posaconazole and voriconazole, only race/ethnicity was significantly correlated with likelihood of achieving therapeutic levels. This is most likely driven by the known CYP2C19 pharmacogenomics variation in voriconazole metabolism [36]. When looking at posaconazole and voriconazole individually, weight normalized TDD was significantly correlated with achievement of therapeutic drug levels. Interestingly, for both antifungals, the non-therapeutic group had a higher median mg/kg/day dose (Tables 2, 3). Possible explanations include serial dose increases for patients with persistently low plasma levels or the saturable absorption for voriconazole and the posaconazole oral suspension [21], evidenced by the low median drug level from posaconazole oral suspension compared to the other formulations in this study. Immunocompetence was significantly correlated with the ability to achieve therapeutic posaconazole levels, but not voriconazole. Another finding of this study was lack of weight being significantly correlated to ability to achieve initial therapeutic posaconazole drug levels. Previous evidence on weight and posaconazole has been conflicting. One study showed no correlation between weight and drug level with the delayed-release posaconazole tablet [37], while another study suggested that patients ≥90 kg had lower mean trough levels than those <90 kg [6].

This is one of the first studies that has looked at all three dosage formulations of posaconazole. The analysis showed the dosage formulation is significantly correlated with ability to achieved a therapeutic plasma level, with the IV formulation having the highest percentage of therapeutic levels (Table 7). When only comparing the oral dosage forms, the delayed-release tablet was sevenfold more likely to achieve a therapeutic level. This is almost identical to the results found in another study comparing the suspension with the delayed-release tablet [37]. Although the delayed-release tablet seemed to predict greater success in achieving therapeutic levels, there are still limitations to its use in patients who are unable to swallow pills whole. Due to this, the critically ill patient population might have a lower percentage of patients able to take the delayed-release tablet. In addition, it can be cost-prohibitive. Several patients in this study switched back to the oral suspension at discharge due to cost.

With more consistent rates of therapeutic levels in this study, there appears to be less PK variability with the IV and delayed-release tablet formulations, indicating TDM might not have the same role for these formulations as for the oral suspension. Although, it is important to note that currently, only the oral suspension has been studied in efficacy trials for the treatment and prophylaxis of invasive aspergillosis [1].

Surprisingly, this study showed a significant correlation between PPI/H2RA use and therapeutic concentrations for both the posaconazole suspension and the delayed-release tablet, while other studies have shown acid suppressing therapies not to significantly affect plasma levels of the delayed-release tablet [37, 38]. Pham et al., however, correlated PPI/H2RA use with median plasma levels, while this study looked at correlation between PPI/H2RA use and achievement of levels >0.7 μg/ml. This highlights some of the present difficulties in studying antifungal TDM and the ability to aggregate the data for clinical decision making.

This study had a large percentage of drug levels that were measured early, which could be a reflection of ICU practices, as nearly half of patients in this study were critically ill. A retrospective study of voriconazole in an ICU reported that 49% of the drug levels measured for TDM were too early [18]. Given the frequency of pre-steady state TDM in real-life ICU settings, we aimed to evaluate the utility of early drug measurements. In this study, the paired analysis showed no significant difference between voriconazole and posaconazole levels pre and post steady-state, indicating that early levels could be predicative of steady-state levels. However, the analysis reflects pooled posaconazole and voriconazole levels. These results must be interpreted in the context of other studies that have analyzed posaconazole and voriconazole individually. For example, data of early dose predictability showed a posaconazole plasma level of ≥0.338 μg/ml on day 3 predictive of a 0.5 μg/ml by day 7 [34]. While other studies have looked at early dose predictability, none of the studies to our knowledge looked at the effect of dose adjustments based on early drug levels. When analyzing dose adjustments, we found that dose adjustments made after a non-therapeutic level, regardless of the timing, tripled the chance of the subsequent level being in the therapeutic range (Table 8).

In this study, non-therapeutic voriconazole levels were associated with adverse drug events (ADEs) requiring discontinuation. Other studies have described how TDM can reduce the rate of voriconazole discontinuation due to ADEs [23, 39]. Unfortunately for this study, it was difficult to accurately assess the total number of adverse drug events requiring discontinuation due to the lack of ADEs reported in patient charts and inability to verify if an ADE was related to voriconazole. Overall, this study adds further support to the use of voriconazole TDM to help prevent ADEs leading to drug discontinuation.

This study did not demonstrate a correlation between survival and therapeutic drug level, which is a finding consistent with the majority of previous studies. Only a few studies have reported a correlation between voriconazole drug concentration and mortality [40–42]. Two of the studies are pediatric studies and the third reported a correlation between a first steady-state trough of voriconazole ≤0.35 mg/l and increased risk of mortality [42]. For posaconazole, it was not surprising to find a lack of correlation between survival and therapeutic drug level in this small sample size, given that a portion of the levels were for prophylaxis rather than treatment.

While many studies have shown a correlation between drug exposure and efficacy [13, 19, 34, 42], in this study neither drug showed a significant difference in treatment failure correlated with therapeutic drug levels. However, voriconazole did show a significant decrease in treatment failure when comparing average drug levels among patients (Table 2). Based on these data, mean drug level might be a more accurate endpoint to predict therapeutic failure. The treatment failure results of this study should be interpreted carefully though, due to the variable follow-up time in assessing if treatment failure had occurred. For many patients, treatment failure was unknown.

In looking at other clinical outcomes, posaconazole demonstrated a significant reduction in length of hospital stay for those who achieved therapeutic levels. Reduction in hospital stay reduces costs and is especially relevant for immunocompromised patients to minimize exposure to hospital acquired infections. Further pharmacoeconomic studies should be conducted to compare the cost-effectiveness of each posaconazole formulation, given the large price variation between them.

This study has many limitations. As a retrospective study, some data, including treatment failure, were difficult to assess based on incomplete or sometimes missing information in patient charts. There was also a selection bias. Although TDM is routinely used for voriconazole and posaconazole at our institution, patients with the greatest concern for PK variability were more likely to receive TDM, resulting in a potentially falsely elevated non-therapeutic rate. With longer treatment durations for fungal infections, this study was also unable to assess the total length of voriconazole and posaconazole therapy, as data on therapy duration outside of the hospital stay was unavailable. Moreover, the study only assessed drug and dose at the time of the drug level measurement. Changes in the drug regimen in response to a non-therapeutic drug level were only seen if a second drug level was measured. Therefore, there were many potential changes to drug therapy that were not assessed in the study. Further, there could have been multiple changes to a patient’s drug regimen between drug levels being drawn that might not be reflected in this study. The study also did not differentiate between treatment and prophylaxis goals for posaconazole. All posaconazole levels were considered therapeutic if >0.7 μg/ml. However, a trough of >1 μg/mL is recommended for treatment in some guidelines [43].

While this study helps add to the evidence regarding TDM utility during posaconazole and voriconazole therapy, it does not address pharmacoeconomic considerations of TDM, evaluate efficacy between dose-adjustment strategies, or elucidate PK differences or TDM utility for treatment of targeted fungal infections. In order to create more comprehensive guidance for best TDM practices, these topics need to be addressed through prospective, randomized trials. In addition, large, prospective, randomized trials are needed to further assess the efficacy of voriconazole and posaconazole TDM on clinical outcomes, including mortality. Although studies suggest potential benefit in decreasing ADE-related drug discontinuation and treatment failure, most of the current studies fail to demonstrate clinical benefit in mortality with TDM.

## Conclusions

In conclusion, the high percentage of non-therapeutic drug levels found in this study supports the benefit of TDM for optimization of drug levels following administration of voriconazole and posaconazole oral suspension in patients at high risk for mortality from fungal infections. The role of TDM for the intravenous and delayed release tablet formulations of posaconazole is less clear due to higher consistency in achievement of goal concentrations with these preparations. Additionally, there could be potential benefit in making dose adjustments from levels drawn prior to steady-state, though further studies are needed to validate these findings.

## Abbreviations

ADE: adverse drug event
CLIA: Clinical Laboratory Improvement Amendments
EORT/MSG: European Organization for Research and Treatment of Cancer/Invasive Fungal Infections Cooperative Group and the National Institute of Allergy and Infectious Diseases Mycoses Study Group
HPLC: high performance liquid chromatography
H2RA: histamine-2 receptor antagonist
IA: invasive aspergillosis
ICU: intensive care unit
IDSA: Infectious Diseases Society of America
IFD: invasive fungal disease
PPI: proton-pump inhibitor
TDM: therapeutic drug monitoring
